# Copyright violation of predators in scientific publishing - Biochemia Medica’s harmful experience and proposed solution

**DOI:** 10.11613/BM.2020.020201

**Published:** 2020-06-15

**Authors:** Daria Pašalić, Vesna Šupak Smolčić

**Affiliations:** 1 Department of Medical Chemistry, Biochemistry and Clinical Chemistry, Zagreb University School of Medicine, Zagreb, Croatia; 2 Clinical Department for Laboratory Diagnostics, Clinical Hospital Centre Rijeka, Rijeka, Croatia; 3 Department of Medical Informatics, Rijeka University School of Medicine, Rijeka, Croatia

**Keywords:** copyright violation, cybercriminal, predatory journal

## Abstract

*Biochemia Medica* is an open access journal that does not charge manuscript processing or publishing. All editorial staff are continuously educated and directed to follow the highest ethical and scholarly publishing standards in all steps of the manuscript processing. They are all laboratory medicine professionals, who apart from their regular jobs, are in charge of different phases in Journal processing as volunteers. The publisher of the Journal is scientific and professional association of laboratory medicine professionals, Croatian Society of Medical Biochemistry and Laboratory medicine (CSMBLM). During November and December 2018, without knowledge of the editorial staff, unknown perpetrator(s) downloaded a respectable number of articles published in *Biochemia Medica* as PDF and launched an illegal web page under the same journal name with downloaded articles. Although this was a very harmful experience, we have learned a lot from it and we would like to share this with scientific journals’ community. Therefore, we would like to share this harmful experience, and to present a short workflow on how to manage situations like this if it will be necessary for any scientific journal in the future.

It is well known that predatory journals do not follow standard polices proposed by the World Association of Medical Editors (WAME). Predatory journals are usually newly established journals that spread their “call for papers” worldwide and charge the publication in open access journals (*1*). It is clear that predatory journals are those who serve for their own financial profit (*2*).

*Biochemia Medica* is an open access journal that does not charge manuscript processing or publishing. All editorial staff are continuously educated and directed to follow the highest ethical and scholarly publishing standards in all steps of the manuscript processing. They are all laboratory medicine professionals, who apart from their regular jobs, are in charge of different phases in Journal processing as volunteers. The publisher of the Journal is scientific and professional association of laboratory medicine professionals, Croatian Society of Medical Biochemistry and Laboratory medicine (CSMBLM).

In practice, each manuscript submitted to the Journal passes several phases during editorial, peer reviewing and publishing process. The steps between the first online submission and the first Editor-in-Chief’s decision are schematically presented in Figure 1. Every publisher, editor, reviewer and author knows that these phases require respectable time and that that time depends on Journal’s policy. The time that passes between online submission of the manuscript and the first Editor-in-Chief’s decision that is made after checking for research integrity issues and peer-review is between 6 to 8 weeks. The rest of the period, prior to manuscript acceptance in the overall timeline depends on the authors. They should, in a timely manner, properly answer to reviewers’ recommendations.

**Figure 1:**
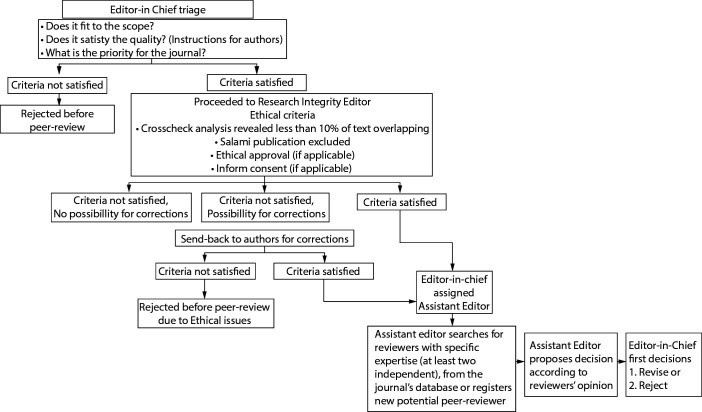
Workflow of the editorial and peer review process for the manuscripts submitted to *Biochemia Medica.*

Research integrity checkup of the manuscript consists of text similarity analysis using Crosscheck plagiarism detection service provided by CrossRef (*3*). Apart from that, research integrity editors also analyze the manuscript for potential salami publication (*4*). Furthermore, each manuscript is evaluated regarding ethical aspects of conducted and presented work (*5*). The final report gives the recommendation whether to proceed with editorial process, to return to the author for smaller corrections and explanation or to reject the manuscript for some of the major breaches of research integrity.

Every editor worldwide knows that the quality of the journal must be continuously improved to be in accordance with recommendations related to ethics, peer-review, online submission system, technical issues, journal web page demands and many others. Every year the editors of *Biochemia Medica* try to identify as many issues to be corrected or improved in order to maintain certain quality level.

In October 2018, after two-year preparation, editorial-publishing office of the journal *Biochemia Medica* has launched new web page because the old one could not compete with modern demands. We have fully transferred all previous, old and recent issues to the new web page. Due to new web page requirements, we changed our server provider and registrar of the domain Biochemia-medica.com. The journal remained open access and free of charge, with copyright transferred to the CSMBLM and with the open-access license under the Creative Commons Attribution 4.0 International License. *Biochemia Medica’s* impact factor for 2017 was 3.653 and the journal was in the Q1 in the category of Medical Laboratory Technology according to Web of science Journal citation reports (JCR).

During November and December 2018, without knowledge of the editorial staff, unknown perpetrator(s) downloaded a respectable number of articles published in *Biochemia Medica* in portable document format (PDF) and launched an illegal web page under the same journal name with downloaded articles. They represented themselves as Journal published by CSMBLM and used the name of Editor-in-Chief to send “call for paper” e-mails with all other relevant information they gathered from our web page in order to get potential authors’ interest. They stated that the journal covered wide area of natural science and biomedicine to attract wider range of authors from different areas. They promised fast peer-review and publication in just a few days from submission. The first suspicion was raised by Journal’s Editor-in-Chief based on the e-mail received at the end of December during Christmas holidays (Figure 2). The day after, one of the Chinese authors who wanted to submit the manuscript to *Biochemia Medica* send an e-mail addressed to the correct e-mail address of the Editorial office with the question about the true domain of *Biochemia Medica*. He wanted to know which of the two, “com” or “org”, was the correct Journal’s web page. Furthermore, he sent us the e-mail received as invitation “call for papers” e-mail with listed contacts. It was obvious that the listed contacts were very similar but not identical to the true Editor-in-Chief’s and Journal’s contacts. As correct suffix was and still is “com”, Senior Editor opened the web page with suffix “org” and revealed the existence of the web page that violated copyright of the *Biochemia Medica*.

**Figure 2:**
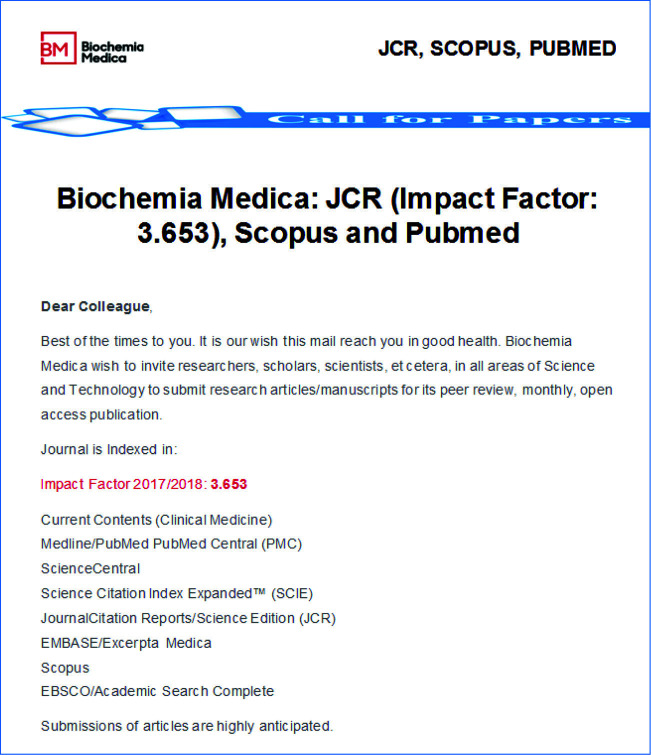
An example of fraudulent e-mail sent to potential authors around the world. The content of the invitation letter was information stolen from *Biochemia Medica’s* web page and therefore violated copyright. E-mail was also sent to the *Biochemia Medica’s* Editor-in-Chief and several editorial-board members.

We contacted immediately our server provider and web page designer who in short time identified the server that hosted the illegal web page and its location in Turkey. They also advised us to contact the Turkish server provider and to inform them about copyright violation by the web page they had hosted. We have kindly asked them to remove illegal web page immediately. In very short time, Turkish server provider did so. We have also contacted the Department for Cyber Criminal of the Ministry of the Interior of the Republic of Croatia and National Computer Emergency Response Team (CERT), an expert group that handles computer security incidents. Furthermore, we have contacted staff from Portal of Croatian Professional and Scientific Journals (HRČAK). All of them advised us how to collect and how to save “original e-mail headers” received from cyber criminals and from the authors who might collaborate with them. As one of the *Biochema Medica*’s Editorial Board member is a professor from one of the Turkish University, we have asked him to help us contact the Turkish Cyber police and Turkish national CERT. Previously listed activities resulted favorable for our Journal because the first server host and illegal web page were removed in two days. However, few hours after that we have noticed that the illegal web page became available again and was hosted by another server provider.

We have contacted again Croatian National CERT, Croatian server provider, our Turkish collaborators and HRČAK. All of them agreed that it will not be enough to contact server providers because it seemed that cyber criminals have a strategy to move from one server provider to another and that we have to contact Registrar which was localized somewhere in India. HRČAK staff helped us to compose a report on our Journal’s copyright violation for acquiring financial gain. In two weeks’ time from the first notification about violation, Registrar blocked the illegal web page.

During this process and later, many potential authors found our original web page and contacts surfing the internet. Some of them were confused by existence of two different Journal web pages and later they were confused when the illegal one terminated but they submitted their manuscripts through contacts available on this web page. All those manuscripts were out of scope and not in compliance with *Biochemia Medica*’s Instructions for authors. Some of the authors sent more than one manuscript. Editor-in Chief answered to each e-mail and informed authors that unknown Cyber criminals deceived them. Some of the authors sent us e-mails received from Cyber criminals including “e-mail headers” as well as the acceptance decision and invoices (Figure 3). Prices were different with the opportunity to arrange a better price deal depending on the author.

**Figure 3:**
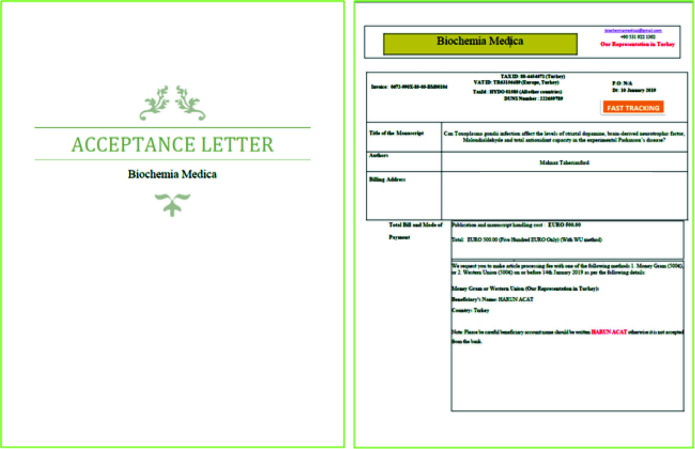
An example of acceptance letter that authors have received few days after manuscript submission. It also included “the invoice”. Received by courtesy of anonymous author.

Although this was a very harmful experience, we have learned a lot from it and we would like to share this with scientific journals’ community. Therefore, we present a short workflow on how to manage situations like this if it will be necessary in the future (Table 1 and Table 2).

**Table 1:** People, services and institutions that can help resolve issues regarding web page theft, cyber-criminal or scientific journal copyright violation

1. Your server provider and Registrar of the domain. Server providers are usually available 24 hours daily. Customer can report suspicious incident at any time. They have competences to help you locating server provides which host illegal web page and Registrars.
2. Web-designers could also be able to do the same as server providers.
3. National CERT: they can give you advice how to download the headers of suspicious e-mails. Those headers might help CERT personal to identify IP addresses of the senders.
4. Cybercriminal office of the Ministry of the interior; report the incident.
CERT - Computer Emergency Response Team. IP – Internet Protocol.

**Table 2:** What you should do to overcome and prevent the problem

1. Save the “headers” of all suspicious e-mails and send them to the National CERT and Cybercriminal team of the Ministry of the interior
2. Report the incident to the Registrar of the domain and server provider. They usually have a link available on their web page through which you can report suspicious activities. Inform yourself about business policy of the Registrar and the ways to prepare the report.
3. Report all illegal e-mail addresses to the relevant e-mail service (Google, Yahoo, Microsoft, Apple, *etc*.)
4. Report spams and phishing e-mails.
5. To prevent similar situation register your web page with more different and overcurrent domains (for example com, org, net, and the one with national extension).
6. Register with your e-mail address at the NormShield service. NormShield will inform you in case someone registers a domain that looks similar to your domain, but you should be aware that this service does not cover the internet space worldwide. NormShield is a cyber-risk rating service that monitors cyber-risk posture of individual or organization through combination of several types of assessment (*6*).
CERT - Computer Emergency Response Team.
